# Farmer perceptions, knowledge, and management of fall armyworm in maize production in Uganda

**DOI:** 10.3389/finsc.2024.1345139

**Published:** 2024-05-17

**Authors:** Thomas Lapaka Odong, Isaac Obongo, Richard Ariong, Stella E. Adur, Stella A. Adumo, Denish Oyaro Onen, Bob I. Rwotonen, Michael H. Otim

**Affiliations:** ^1^ Department of Agricultural Production, Makerere University, Kampala, Uganda; ^2^ National Crops Resources Research Institute–Namulonge, National Agricultural Research Organization, Kampala, Uganda; ^3^ Development Strategy and Governance Division, International Food Policy Research Institute, Kampala, Uganda; ^4^ National Agricultural Research Laboratories, Kawanda, National Agricultural Research Organization, Kampala, Uganda

**Keywords:** awareness, damage, information channels, insecticide, yield loss, *Spodoptera frugiperda*

## Abstract

*Spodoptera frugiperda* (J.E. Smith), fall armyworm (*FAW*), a polyphagous Noctuid pest, was first reported in Uganda in 2016. Farmers were trained to identify and manage the pest, but there was a lack of information on farmer knowledge, perceptions and practices deployed to control it. Therefore, we conducted a survey to assess maize farmers’ knowledge, perceptions and management of the pest during the invasion. We interviewed 1,289 maize farmers from 10 maize-growing agro-ecological zones (AEZ) of Uganda using well-structured questionnaires. The data were analyzed using R version 4.2.3. The respondents faced many constraints, including pests, drought, poor soils and labor constraints. Among the pests, *FAW* was ranked by most (85%) of the respondents as the number one pest problem in maize, and some farmers reported having noticed it way back in 2014. By 2018, more than 90% of the farmers had seen or heard about *FAW*, and about 80% saw *FAW* in their fields. The most common *FAW* symptoms reported by maize farmers were windowing, near tunnel damage, and holes on the cobs. The developmental stages of *FAW* identified by farmers included eggs (10%), young larvae (78.7%), mature larvae (73.5%) and adult moths (6.7%). Insecticides were the major control tactic, although some farmers used plant extracts, hand-picking, sand, and ash. Farmers sourced information on *FAW* from various sources, including fellow farmers, radio/TV, extension agents, input dealers, print media, research and NGO extension. There is a need to package clear and uniform information for the farmers and to develop and promote a sustainable solution for *FAW* management, including harnessing biological control and cultural practices.

## 1 Introduction

Maize (*Zea mays*) is the leading crop in terms of global production among all cereal crops (1). It is a source of income, food rich in carbohydrates, fibre, and protein, and some varieties are rich in carotenoids (2). Maize is mainly grown by small-scale farming households at the subsistence level in sub-Saharan Africa. The production of maize in Africa is constrained by abiotic factors, which include drought and water shortage (3) and biotic factors. The biotic factors include weeds, especially *Striga* spp (major parasitic weed of cereals) (4), and insect pests such as stemborers (5–8) and, more recently, the fall armyworm (*FAW*) *Spodoptera frugiperda* (J.E. Smith) (Lepidoptera: Noctuidae) (9). The *FAW* was first reported in Africa in 2016 (10) and had spread to 50 African and 25 Asian countries by October 2023 (11). In Uganda, *FAW* was first observed in 2016 in experimental fields and spread to all the maize-growing areas within a year (12). *Spodoptera frugiperda* spread rapidly on the African continent and beyond because of its high migratory ability, wide range of host crops, high reproductive potential, and preference for maize (9, 13, 14).

*Spodoptera frugiperda* undergoes complete metamorphosis, with a life cycle duration of about 30 days (14). On maize, first instar larvae usually feed on the epidermal tissue of leaves, creating a characteristic windowing effect. The second or third instar, larvae eat leaves, making holes in them (15). Feeding in the funnel of maize plants often produces a characteristic row of perforations in the leaves. Older larvae cause extensive defoliation, often leaving only the ribs and stalks of maize plants or a ragged, torn appearance. Feeding by older larvae leaves moist sawdust-like frass near the funnel and upper leaves. In young plants, the stem may be cut. Later, the larger larvae can enter maize cobs, reducing yield quantity and quality (15). Such signs and symptoms are key for the farmers to correctly identify and institute the right control measures.

Many authors estimated huge losses attributed to this pest based on the American experience. For example, yield losses of 8.3 to 20.6 million tons of maize, valued yearly at USD 2,481–6,187 million, were estimated for Africa (16). The annual loss for Uganda was estimated at 558.9 to 1,391 tons under no control, which translated to USD 163.7 to 407.5 million annually (16). In Zambia, *FAW* was reported to have affected about 130,000 hectares of maize and resulted in over USD $3 million for control costs during the early stages of its introduction (http://www.fao.org/africa/news/detail-news/en/c/469532/). The presence of *FAW* in Africa resulted in panic among international and national research organizations, Governments and farmers, among others. In Uganda, like many other African countries, the *FAW* invasion led to the registration, purchase and distribution of insecticides for its control.

Integrated pest management has been fronted as the most economical and more benign strategy to manage *FAW* in Africa (9). In order to design an acceptable pest management strategy, it is vital understand farmers’ basic socio-economic characteristics, knowledge and perceptions, coping measures and constraints to effective implementation of the pest management practices. Despite the efforts to control *FAW* in Uganda, the lack of information on farmers’ perceptions, knowledge, behavior, and coping strategies limits the development and promotion of sustainable (effective and acceptable) solutions to the pest (12). Since its introduction, a few selected studies documented farmers’ perceptions and management of the pest in Uganda (17, 18), both of which focused on a limited geographic scope. This study sought to document: i) major constraints faced by farmers; ii) farmers’ awareness and knowledge of *FAW*; iii) maize grain yield losses; iv) practices used by farmers to control *FAW* and their perceived effectiveness; v) information sources on *FAW.* We chose to conduct the study in different agro-ecological zones (AEZ) because of the known differential influence of environmental and human factors on the incidence, damage and response of *FAW* to control. The different AEZs are a representation of different maize production environments within the country.

## 2 Materials and methods

### 2.1 Study area

**Figure 1:**
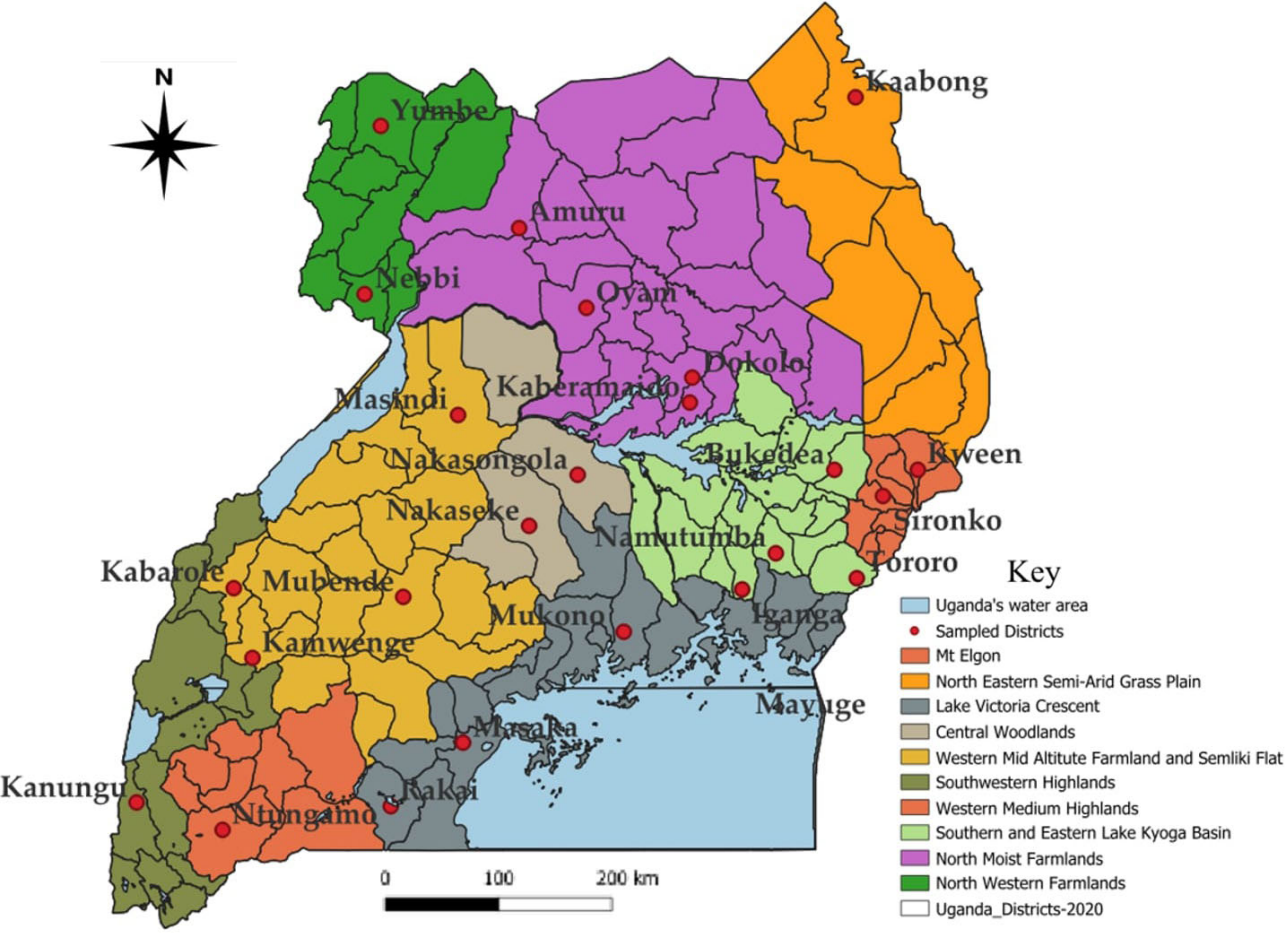
Map of Uganda showing the study districts and agro-ecological zones where the surveys were conducted.

The study was conducted in 25 districts selected from all the nine zones of Uganda adopted based on the National Agricultural Research Organization (NARO) zoning, namely Buginyanya, Abi, Kachekwano, Mbarara, Mukono, Nabuin, Bulindi, Ngetta and Rwebitaba (**Table 1**; **Figure 1**). These zones generally produce maize at varying levels due to differences in relative importance attached to the crop in the different regions. Uganda experiences two rainfall seasons annually. The first rainy season run from March to early June (denoted as the “first season or season A”) and the second rainfall season majorly running from August to December (denoted as the “second season or season B”).

**Table 1:** Sample size distribution in the different agro-ecological zones.

	Agro-ecological zones of Uganda	Number of sampled districts	Sample size (n)
1	Central Woodlands (CW)	2	102
2	Lake Victoria Crescent (LVC)	4	203
3	Mount Elgon	2	95
4	Northeastern Semi-Arid Grass Plain (NeSASGP)	1	31
5	North Moist Farmlands (NMF)	4	194
6	Northwestern Farmlands (NwF)	2	100
7	Southern and Eastern Lake Kyoga Basin (SELKB)	4	183
8	Southwestern Highlands	1	77
9	Western Mid-Altitude Farmlands and Semiliki Flats (WMAFSF)	4	242
10	Western Medium-High Farmlands (WMHF)	1	62
	**Total**	**25**	**1,291**

### 2.2 Sample selection

A combination of a three-stage stratified sampling procedure and the probability proportionate to population method was used to cluster respondents at the three levels of zones, districts and sub-counties. The study sites were grouped into nine blocks/strata (L = 9) based on the NARO agricultural research zones mentioned above. These included Central Woodlands (CW), Lake Victoria Crescent (LVC), Mount Elgon, Northeastern Semi-Arid Grass Plain (NeSASGP), North Moist Farmlands (NMF), Northwestern Farmlands (NwF), Southern and Eastern Lake Kyoga Basin (SELKB), Southwestern Highlands, Western Mid-Altitude Farmlands and Semiliki Flats (WMAFSF) and Western Medium-High Farmlands (WMHF).

Districts were stratified based on the nine NARO agro-ecological zones, and the number of districts per zone was computed based on the probability weight in each zone. After determining the number of districts to be selected per AEZ (at least two), we applied a simple random sampling technique to select specific districts for the survey, leading to a sample of 29 districts being selected based on the weighted probabilities. In each ***s*** elected district, two sub-countries were purposely selected based on maize production levels. A total of 26 farmers from one randomly sampled village per sub-county were targeted for interview. Overall, 1,291 respondent households were targeted for interview. Stratified sampling was used with unequal proportion due to significant differences in the size of the stratum (Equation 1) (19).


(1) $$n_{h}=\left(\frac{N_{h}}{N}\right)n$$

Where: *n_h_* is the sample size in stratum *h* and *h* = 1,2,…,*L* while *N_h_* is the size of the population stratum *vh*. *N* Denotes the total population size, which is a summation of the population size per stratum ( $N=\sum_{h=1}^{L}N_{h}$ ) and *n* is the total sample size ( $n=\sum_{h=1}^{L}n_{h}$ ). Proportional allocation was adopted because it minimizes the variance of the estimators, and thus, zones with larger populations, which may be associated with higher variability, will require more sample units to attain the same degree of precision as in zones with smaller populations. Thus, the larger the stratum population *N_h_*, the larger the required sample *n_h_*. Considering a *z* score of 1.96, margin of error *d* = 0.05, the strata number *L* = 9 and the stratum populations obtained from the Uganda Bureau of Statistics census report 2014 (20), the total sample size needed with proportional variation is derived from the expression in Equation 2 (19). Note that variance of stratum $\sigma_{h}^{2}$ is derived from the formula $\sigma_{h}^{2}=\frac{N_{h}}{N_{h}-1}p_{h}\left(1-p_{h}\right)$ .


(2) $$n=\frac{Z^{2}}{d^{2}}\sum_{h=1}^{L}\left(\frac{N_{h}}{N}\right)\sigma_{h}^{2}n=1,291$$

The stratum sample population *n* is a total sum of strata populations shown in **Table 1**. The number of households per district was fixed at 53, while the number of districts per zone is based on probability weights.

### 2.3 Data collection and survey instruments

Data were collected from only 1,289 out of the sample target of 1291 maize farmers. The data collection took place during the month of May/June 2018. Data were collected using tablet-based semi-structured questionnaire via the CTO platform (Survey CTO, Doblity, Cambridge, USA; https://www.surveycto.com), which is an Android app that is used to design survey questionnaires and carry out data collection, storage, and monitoring processes from within a unified platform. The data questionnaire covered the socio-economic characteristics of the farmers, constraints faced in maize production, when *FAW* was first seen, stages and symptoms observed by farmers, *FAW* coping measures, the sources of information on *FAW* and yield of maize in the different seasons. The questionnaire was administered by research scientists and technicians after training and pre-testing the questionnaire for its validity and in farmers’ respective local languages. All field data were transmitted to the central server (account) for cleaning and consolidation prior to analysis. The initial target was 29 districts, but by the time of the study, farmers in four districts had already harvested their maize, and yet this was part of a bigger study that involved field assessment of maize plants in the field. So, we ended up with 25 districts.

### 2.4 Data analysis

Data were analyzed using R version 4.2.3 (21). Both descriptive and inferential statistics were used in the analysis. Chi-square was used to study associations between different variables, namely: AEZs and maize production constraints, AEZ and *FAW* awareness, AEZs and *FAW* symptoms/signs observed, AEZs and practice to control *FAW*, AEZs and *FAW* management information sources. The counts and proportion of the respondents for the different variables were obtained and used for constructing different graphs. The number of farmers who saw *FAW* in all the years was summed up to obtain the cumulative proportion.

A generalized linear model was used to analyze perceived yield loss, and the mean losses for the different AEZs were compared using Fisher’s protected LSD at a 5% significance level.

The order of importance of maize production constraints was determined using a weighted score. The weighted score of each constraint was obtained by multiply the weight given to each rank (Weight from rank 1 = 3; weight for rank 2 = 2 and weight for rank 3 = 1) given to each constraint by farmers and the number of farmers who gave the constraint that particular rank and this summed over all the ranks.


$$Wscore=\sum_{i=1}^{3}WR_{i}\;*\;freq_{i}$$

Where WScore = weighted score; *WR* = weight given to rank i and *freq_i_* = frequency of farmers who gave the constraint rank i.

## 3 Results

### 3.1 Socio-demographic characteristics of the interviewed farmers

The respondents comprised male and female farmers of different age groups. The majority (72.4%) of the maize farmers were male. About 90% of farmers were below 60 (range, 18 – 93 years), while most of them were between 30 and 49 years old (**Table 2**).

**Table 2:** The distribution of respondents in the different agro-ecological zones by age groups.

Agro-ecological zone*	Age groups								Proportion per AEZ (%)
	< 20	20–29	30–39	40–49	50–59	60–69	70 and above	Grand total	
**CW**	0	12	25	38	20	5	2	102	7.9
**LVC**	0	14	69	68	29	16	5	201	15.6
**Mt. Elgon**	0	16	26	26	13	8	6	95	7.4
**NeSASGP**	0	10	15	5	0	1	0	31	2.4
**NMF**	1	29	62	48	35	16	3	194	15.1
**NwF**	1	23	27	27	14	7	1	100	7.8
**SELKB**	0	20	41	54	41	22	5	183	14.2
**SwH**	0	11	20	26	16	4	0	77	6.0
**WMAFSF**	0	41	72	53	49	23	4	242	18.8
**WMHF**	0	8	20	18	12	4	0	62	4.8
**Grand Total**	**2**	**184**	**377**	**363**	**229**	**106**	**26**	**1,287**	
**Proportion per age group (%)**	**0.16**	**14.30**	**29.29**	**28.21**	**17.79**	**8.24**	**2.02**		

CW, Central Woodlands; LVC, Lake Victoria Crescent; NeSASGP, Mount Elgon, Northeastern Semi-Arid Grass Plain; NMF, North Moist Farmlands; NwF, Northwestern Farmlands; SELKB, Southern and Eastern Lake Kyoga Basin; WMAFSF, Southwestern Highlands, Western Mid-Altitude Farmlands and Semiliki Flats; WMHF, Western Medium-High Farmlands.

### 3.2 The education level of the interviewed farmers

Most of the farmers attended formal education, with more than half (52.4%) of them reaching primary level and slightly above a quarter attaining secondary level education. Nevertheless, 6.2% of the farmers did not attend any formal education (**Table 3**).

**Table 3:** Proportion of respondents from different agro-ecological zones who attained different levels of education.

Agro-ecological zones	N	Junior level	No formal education	Primary level	Secondary O’ level	Secondary A’ level	Tertiary certificate	Acquired diploma	University
CW	102	0.0	2.9	57.8	32.4	2.0	2.9	1.0	1.0
LVC	201	0.5	4.0	55.7	25.4	2.5	2.5	4.5	5.0
Mt. Elgon	95	0.0	1.1	45.3	37.9	3.2	6.3	4.2	2.1
NeSASGP	31	0.0	19.4	48.4	19.4	3.2	6.5	3.2	0.0
NMF	194	1.0	3.6	54.6	26.8	4.6	8.8	0.5	0.0
NwF	100	2.0	9.0	42.0	25.0	4.0	12.0	4.0	2.0
SELKB	183	0.5	8.2	46.4	27.9	6.6	2.2	5.5	2.7
SwH	77	0.0	6.5	68.8	18.2	2.6	2.6	1.3	0.0
WMAFSF	242	1.2	8.7	51.7	26.4	2.9	4.5	2.1	2.5
WMHF	62	0.0	8.1	56.5	12.9	9.7	8.1	1.6	3.2

### 3.3 Constraints to maize production

Farmers ranked pests, drought, poor soils, and labor as the most important constraints in maize production overall in order of importance (**Table 4**). The ten most important production constraints in maize production fall within the broad categories of biotic, abiotic and socio-economic factors. Out of these ten major constraints, three are biotic, falling as number 1 (pests), 7 (striga/the witchweed) (Striga spp.) and 9 (vermin (Rodents). Four of the constraints are abiotic, ranked as number 2 (drought), 3 (poor soils), 5 (unpredictable rains), and 10 (excessive rains/floods). The socio-economic ones were three being, ranked as 4 (labor constraints), 6 (limited access to quality seed) and 8 (limited access to fertilizers and pesticides). Other constraints included limited access to agrochemicals, flooding, lack of knowledge and information, and lack of access to extension services. Interestingly, some farmers did not state that they faced production constraints. Save for pests and drought, which ranked first and second in most agro-ecologies, there were some variations in the ranking of the other constraints in the different agro-ecologies. For instance, labor shortage was the third most important constraint in four of the Agro-ecological zones, and vermins and Striga weed the second most important in the NeSASG and SELKB AEZs, respectively.

**Table 4:** Weighted scores for constraints that farmers reported to have affected maize production in the different agro-ecological zones from 2014 to 2017.

Constraint	CW (n=102)	LVC (n=203)	Mt. Elgon (n=95)	NeSASGP (n=31)	NMF (n=194)	NwF (n=100)	SELKB (n=183)	SwH (n=77)	WMAFSF (n=242)	WMHF (n=62)	Weights (n=1289)
Pests	233	418	226	67	488	217	423	152	413	104	2741
Drought	160	252	112	29	201	192	152	192	523	142	1955
Poor soils	24	180	57	1	22	37	79	36	131	56	623
Labour constraints	67	88	23	2	89	25	56	46	166	26	588
Unpredictable rains	8	58	32	13	63	40	43	12	39	14	322
Limited access to quality seed	41	48	14	5	79	40	27	0	59	2	315
Striga	4	14	0	0	45	8	184	0	0	0	255
Limited access to fertilizers and agro-chemicals	21	51	26	0	8	8	30	4	21	0	169
Vermin	17	7	0	49	10	0	5	0	18	1	107
Excess rains/floods	1	6	31	15	11	1	23	0	12	4	104
Diseases	3	11	20	0	16	2	19	0	0	10	81
Theft	2	13	0	0	8	3	8	8	8	5	55
Limited access to knowledge & information	1	4	2	0	13	1	6	2	3	0	32
Limited access to extension services	0	2	0	0	6	0	0	2	2	0	12
None	30	66	27	5	105	26	43	8	57	8	375

### 3.4 Major pests reported by farmers in different agro-ecological zones

Farmers listed four pests as affecting maize production in their localities. These include fall armyworm, termites and stemborers (**Table 5**). The *FAW* was ranked as the most important insect pest by the majority of farmers from all the agro-ecologies, followed by termites (3.5%), stemborers (2.7%), and monkeys (1.5%). Termites were reported by a relatively higher proportion of farmers in NMF (11.3%), and CW (8.8%) compared to other agro-ecologies. On the other hand, stemborer was relatively more important in NwF (9.0%) and NMF (8.8%) compared to other agro-ecologies (**Table 5**).

**Table 5:** Number and proportion of farmers who reported different pest problems between 2014 and 2018 in the different agro-ecological zones of Uganda.

Agro ecologies	Total number (n)	Fall armyworm (%)	Termites (%)	Stemborers (%)	Monkeys (%)
CW	102	84.3	8.8	2.9	3.92
LVC	203	83.7	4.4	0.0	2.46
Mt. Elgon	95	90.5	2.1	2.1	0.00
NeSASGP	31	90.3	0.0	0.0	0.00
NMF	194	80.9	11.3	8.8	2.06
NwF	100	85.0	2.0	9.0	3.00
SELKB	183	91.3	2.2	1.1	0.55
SwH	77	93.5	1.3	0.0	0.00
WMAFSF	242	74.8	2.5	2.9	1.65
WMHF	62	74.2	0.0	0.0	1.61

### 3.5 Awareness of *Spodoptera frugiperda* in the different agro-ecological zones

**Figure 2:**
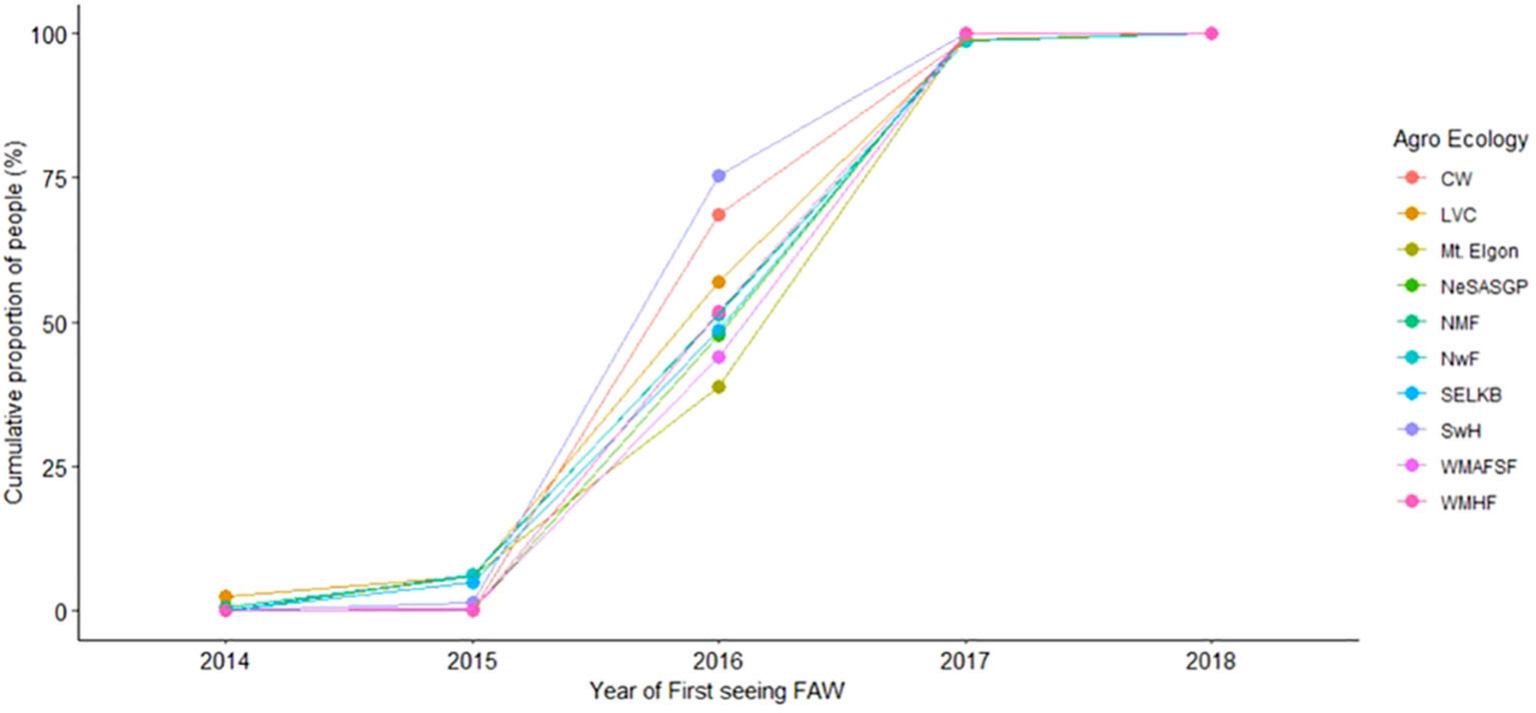
Cumulative proportion of farmers and the year when they first saw *Spodoptera frugiperda*, from 2014 - 2018.

**Figure 3:**
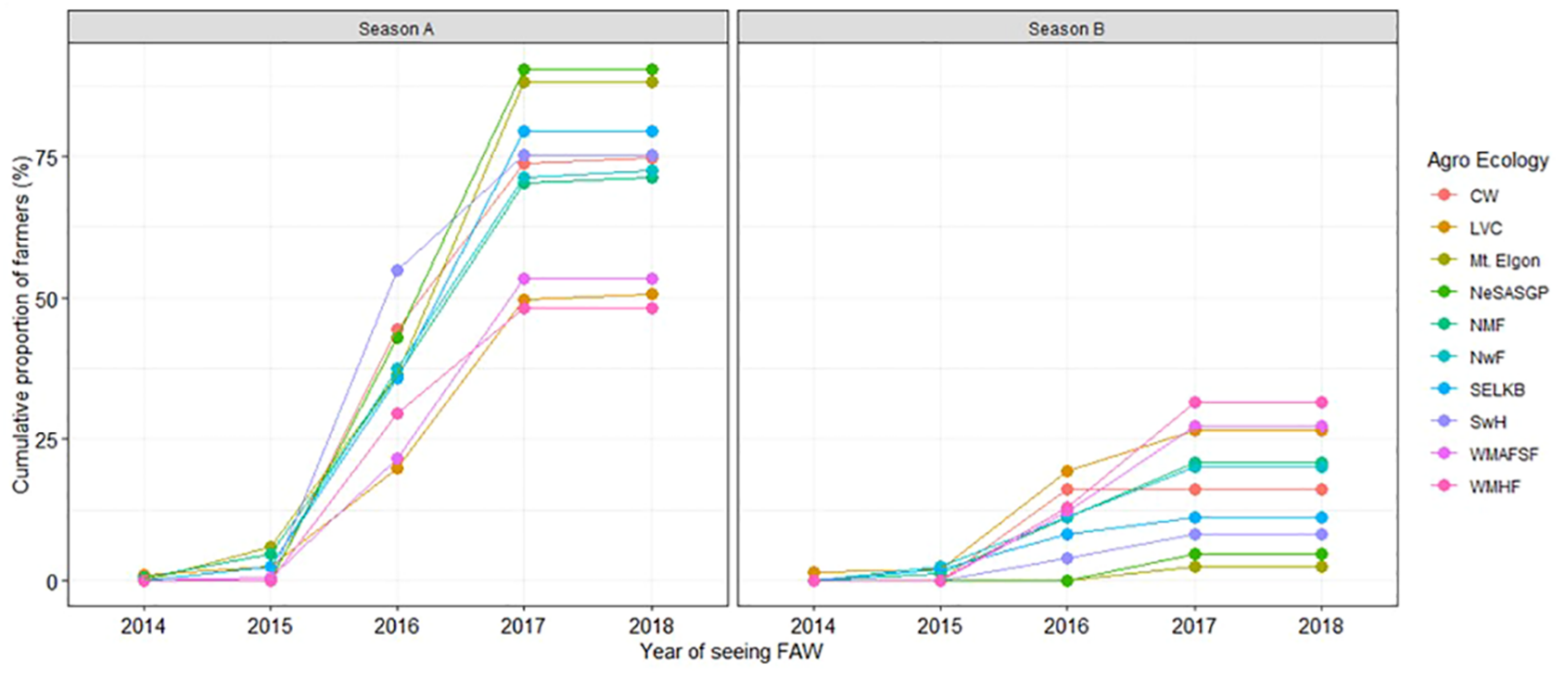
Cumulative proportion of maize farmers who saw *Spodoptera frugiperda* in their own fields by season.

The proportion of farmers who saw *FAW* increased from 2014 to 2018 when nearly all of them had seen the pest/its damage (**Figures 2** and **3**). In all agro-ecologies, significantly (t=8.086, p=1.07211e-05) more farmers first observed *FAW* in the first season (79.4%) compared to the second season (20.1%) (**Figure 3**).

### 3.6 Identification of stages of *Spodoptera frugiperda*

**Figure 4:**
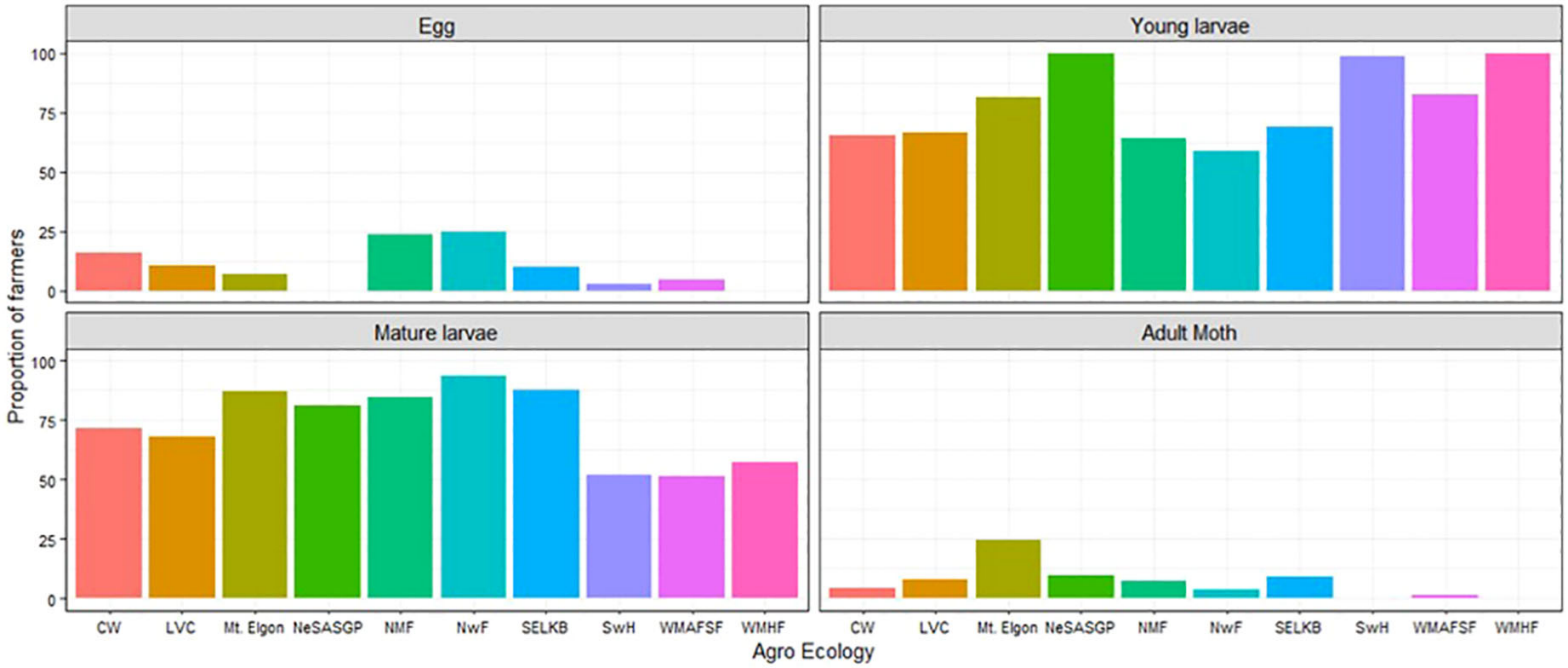
Proportion of farmers who saw different developmental stages of *Spodoptera frugiperda* in the different agro-ecological zones of Uganda, 2018.

The *FAW* developmental stages observed by maize farmers varied significantly among the different AEZs (p< 2.2e-16). At least 50% of farmers observed mature (bigger) larvae and young (smaller) larvae from the different AEZs of Uganda. A small proportion of less than 26% observed the eggs and the adult moths in the different AEZs (**Figure 4**). In NeSASGP and MWHF, none of the farmers reported seeing the eggs of *FAW*. The same was the case for adults in SwH and MWHF (**Figure 4**).

**Figure 5:**
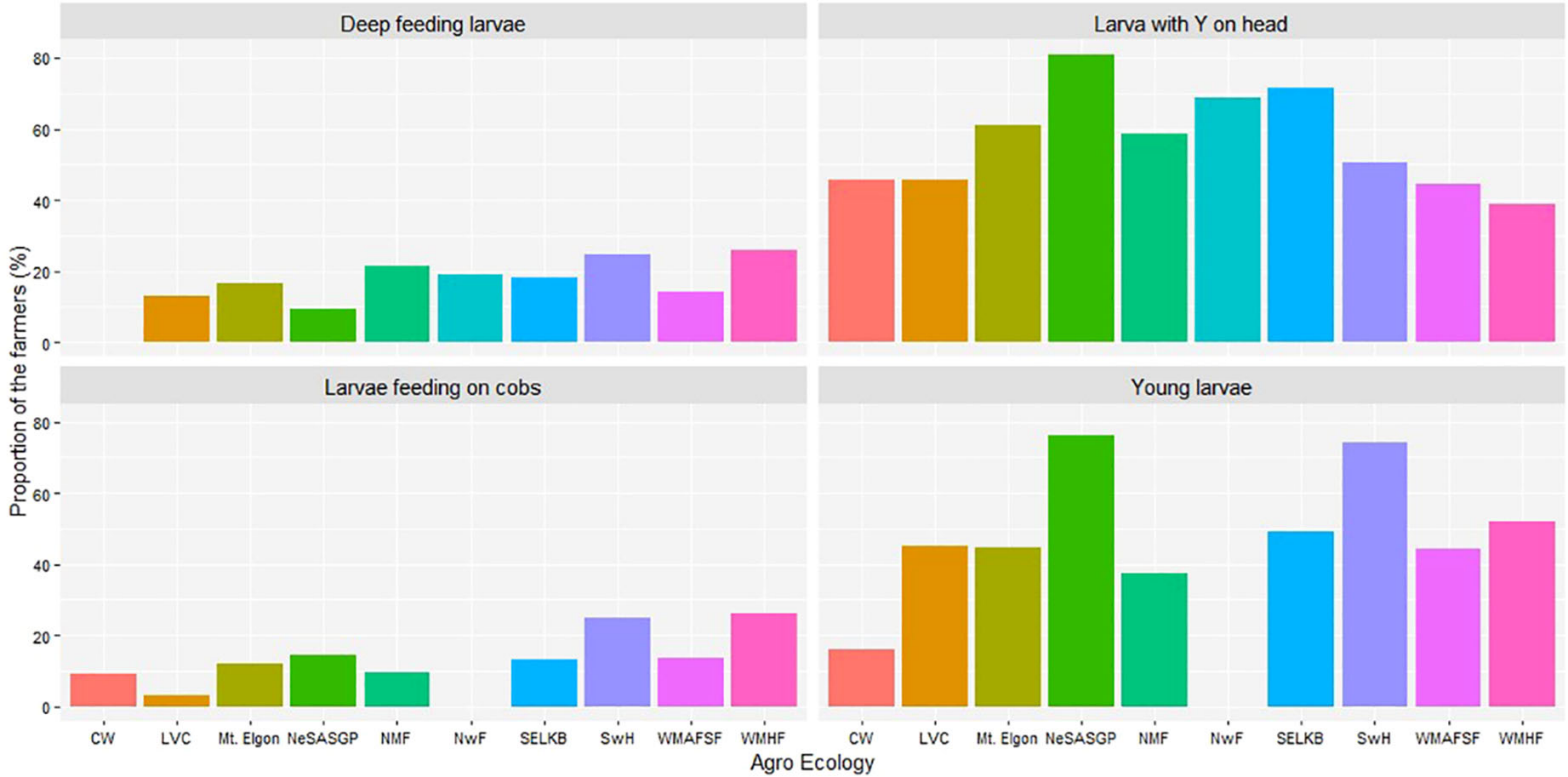
Proportion of farmers who reported different signs of *Spodoptera frugiperda* in the different agro-ecological zones of Uganda, 2018.

The maize farmers were able to identify the different stages of *FAW* larvae (**Figure 5**). The most commonly seen larvae were those with Y-shape on the head, with at least 40% of farmers from the different AEZs identifying them. The other stage was the young larvae, seen by at least 30% of farmers from most of the AEZs except for CW and NwF with at most 18% and 0.0%, respectively. The deep-feeding larvae and larvae feeding on cobs were only noticed by a few or no farmers, depending on the AEZ. Farmers from CW never reported seeing deep-feeding larvae, while none of those in NwF saw larvae on cobs.

### 3.7 Severity of *Spodoptera frugiperda* damage

**Figure 6:**
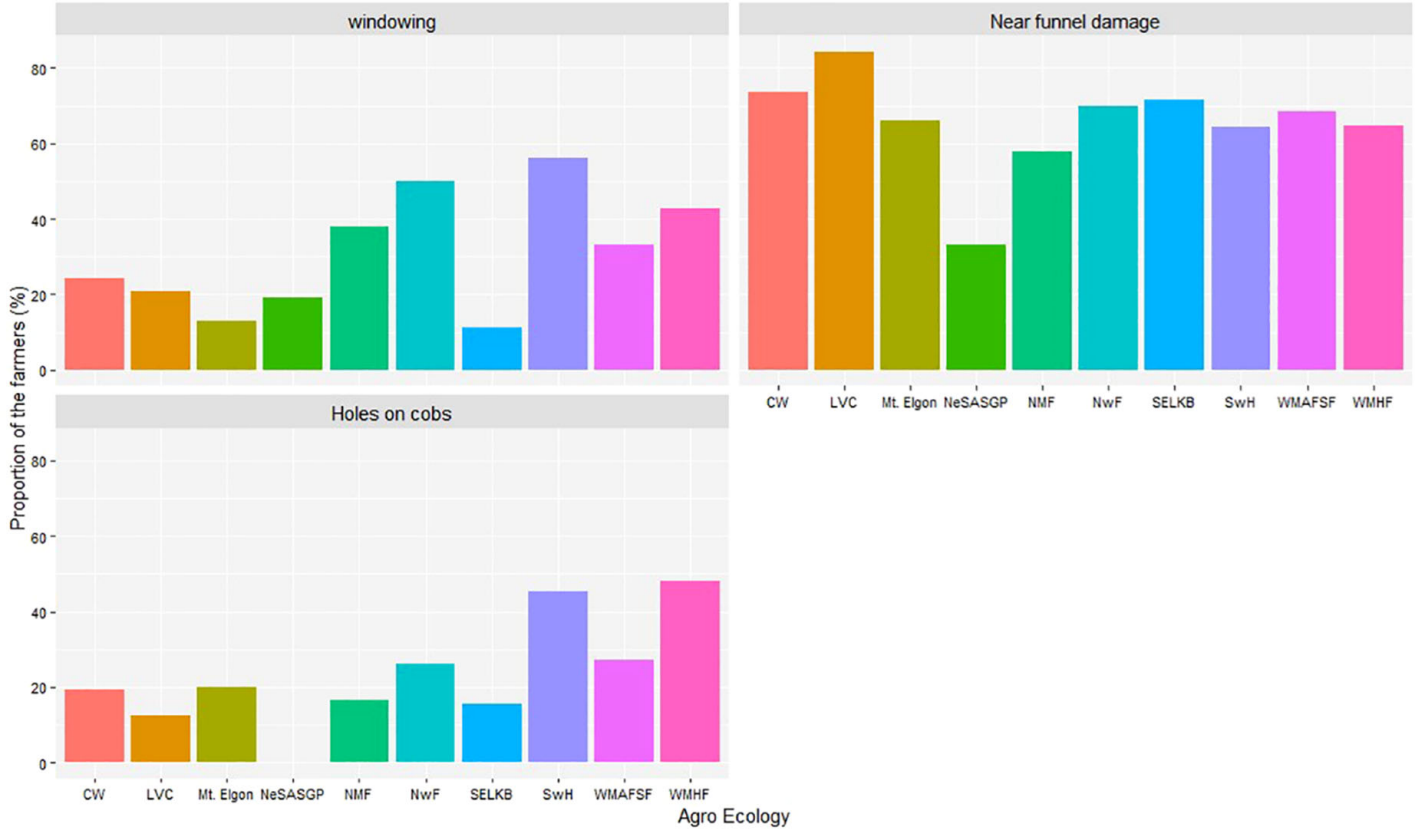
Proportion of farmers who reported different signs and symptoms of *Spodoptera frugiperda* in the different agro-ecological zones of Uganda, 2018.

The proportion of farmers who recognized or identified the different symptoms and signs associated with *FAW* are indicated in **Figure 6**. In decreasing order, the most commonly reported symptoms were damage near the funnel, windowing, and holes on the ear.

**Figure 7:**
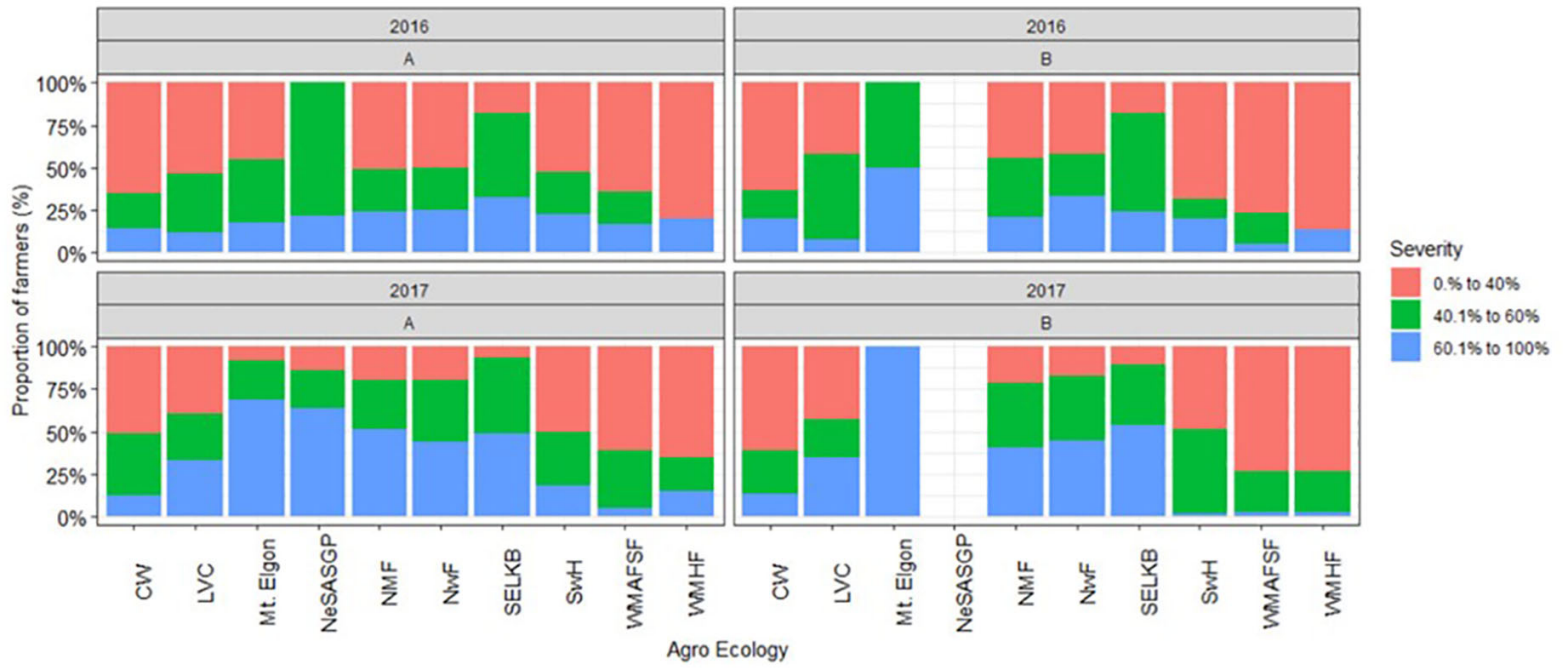
Proportion of farmers who reported different severity levels of *Spodoptera frugiperda* in the different agro-ecological zones of Uganda, 2018.

The severity of *FAW* damage varied among the AEZs and seasons (**Figure 7**). In 2016A, many of the respondents in most AEZs reported lower severity levels (0–40%), except in SELKB. The severity levels increased over time, and more severe symptoms (60–100%) were reported by many farmers in four of the nine AEZs in 2017A. In 2017B, All the farmers in the Mt Elgon AEZ reported severe symptoms (60–100%).

### 3.8 Estimated grain yield losses derived from farmers’ recall of obtained yields

The grain yield losses varied significantly between AEZs in the two seasons of 2016 and 2017B (**Table 6**). In 2016A, NwF had the highest loss of 49.1%, while CW had the lowest loss of 19.5%. In 2016B, SELKB had the highest loss of 44.1%, while WMAFSF had the lowest loss of 9.4%. In 2017B, NwF had the highest loss of 66.9%, while WMAFSF had the lowest loss of 10.7%.

**Table 6:** Derived yield losses in the different Agro-ecological zones and seasons, and FAW damage severity across seasons.

Agro ecology	Percentage yield loses in season		
	2016A	2016B	2017B
**NwF**	49.05^a^	43.22^ab^	66.88^a^
**SwH**	43.13^ab^	30.58^b^	15.67^bc^
**WMHF**	39.26^abc^	39.07^ab^	30.42^b^
**NMF**	39.08^abc^	41.84^ab^	38.65^b^
**SELKB**	35.92^bc^	44.13^a^	44.00^b^
**NeSASGP**	33.61^bcd^		
**LVC**	32.45^bcd^	35.51^ab^	30.38^b^
**Mt. Elgon**	26.69^cd^	14.83^bc^	48.02^ab^
**CW**	19.54^d^	30.52^b^	34.49^b^
**WMAFSF**	19.04^d^	9.43^c^	10.69^c^
**Overall mean**	**31.6**	**30.8**	29.6
Severity			
**60.1% to 100%**	43.21^a^	37.92^a^	40.02^a^
**40.1% to 60%**	36.00^ab^	36.90^a^	32.87^ab^
**0% to 40%**	29.40^b^	29.46^a^	24.01^b^

For each season and parameter, means within a column followed by different letters are significantly different at *p* < 0.05.

The yield losses varied significantly among the different *FAW* damage severity, with the severity of 60.1% to 100% having the highest loss of 39.0%, followed by 40.1% to 60% with 32.76% and finally, 0.0% to 40% having the lowest yield loss of 27.3%. Season-wise comparisons showed that in 2016A, there was a significant difference among the different severity categories, with the highest loss of 43.2% incurred for the severity of 60.1% to 100% and no significant differences among the different severity in 2016B. In 2017B, there was a significant difference among the different severity (**Table 6**).

### 3.9 Management of *Spodoptera frugiperda* in the different agro-ecological zones

**Figure 8:**
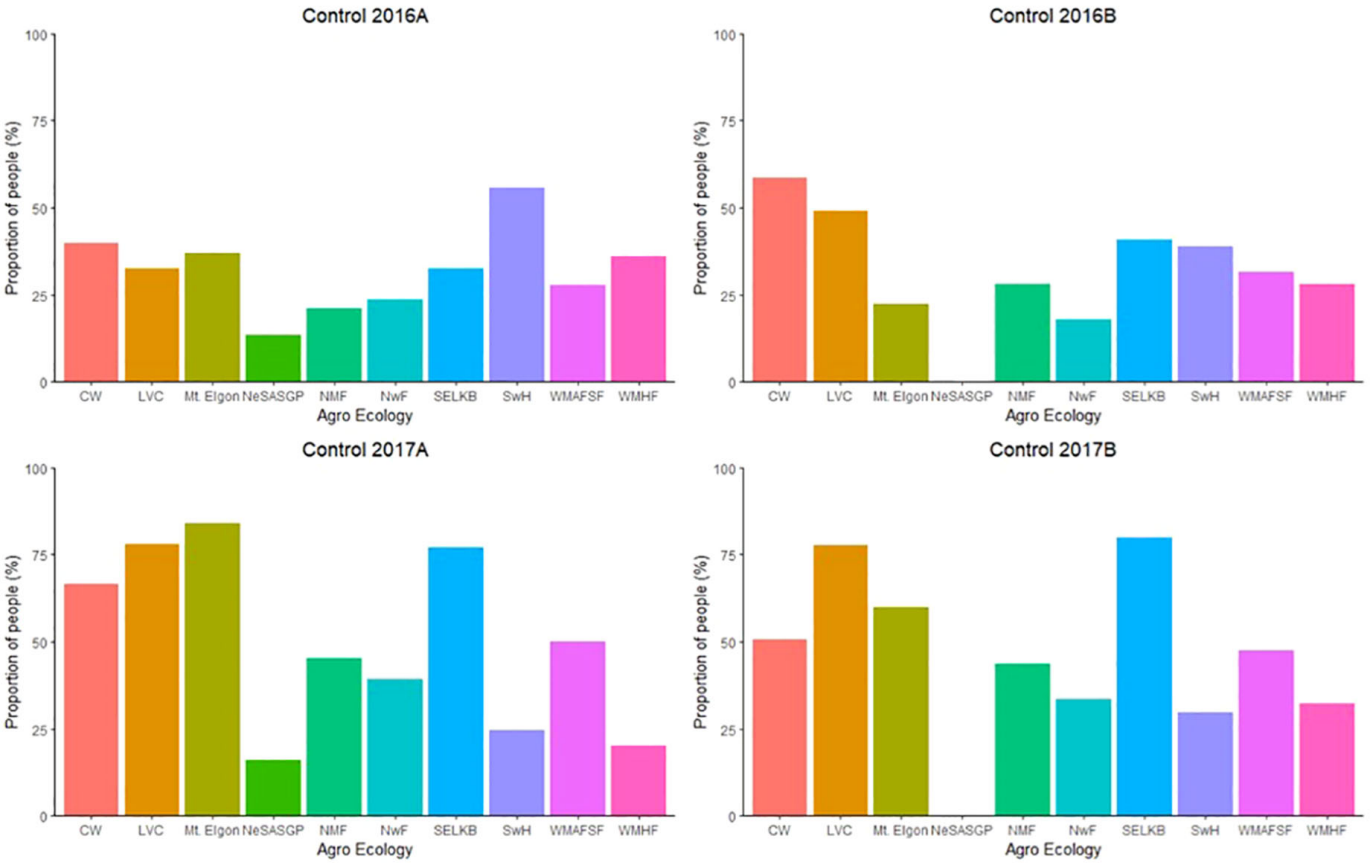
Proportion of farmers who took the initiative to control *Spodoptera frugiperda* in the different agro-ecological zones of Uganda, from 2016 to 2017.

The proportion of farmers who took initiative to manage *FAW* varied among the different AEZs (**Figure 8**). The proportions of farmers who made effort to control *FAW* ranged from 13.3% in NeSASGP to 55.7% in SwH in 2016A, 0 in NeSASGP to 58.6% in CW in 2016B, 16.1% in NeSASGP to 84.0% in Mt. Elgon in 2017A, and 0 in NeSASGP to 79.9% in SELKB in 2017B.

Of the farmers who controlled *FAW* in their fields, an average of 91% used synthetic insecticides (**Table 7**). These ranged from 88.7% in 2016A to 93.6% in 2016B. The other methods used by the farmers included plant extracts, hand picking, use of sand, ash and a change in the cropping system. An average of 76.5% of the farmers who applied insecticides used Cypermethrin-based products.

**Table 7:** The percentage of control measures applied by some farmers in the different AEZs in 2016 and 2017.

Control measures	2016A n=204	2016B n=248	2017A n=574	2017B n=465
Sprayed with insecticide	88.7	93.6	92.5	90.6
Plant extracts	2.0	0.8	0.2	0.7
Hand picking	3.9	2.0	1.4	1.7
Sand	0.0	0.0	0.7	0.4
Applied ash	1.5	0.8	1.9	2.6
Change in cropping pattern	0.0	0.4	0.0	0.4
Other	3.9	2.4	3.3	3.4

Among all the farmers who took the initiative to control *FAW*, 2.4% and 1.7% from NMF reported failing to cope with *FAW* in 2014A and B, respectively. In 2015A, 2.4% of farmers in NwF mentioned not being able to cope with *FAW*, and this number increased to 20.0% and 21.4% in 2016A and 2016B, respectively. The highest proportion of farmers (33.3%) failed to cope with *FAW* in 2016B in the SwH.

### 3.10 Sources of information on *Spodoptera frugiperda*

**Figure 9:**
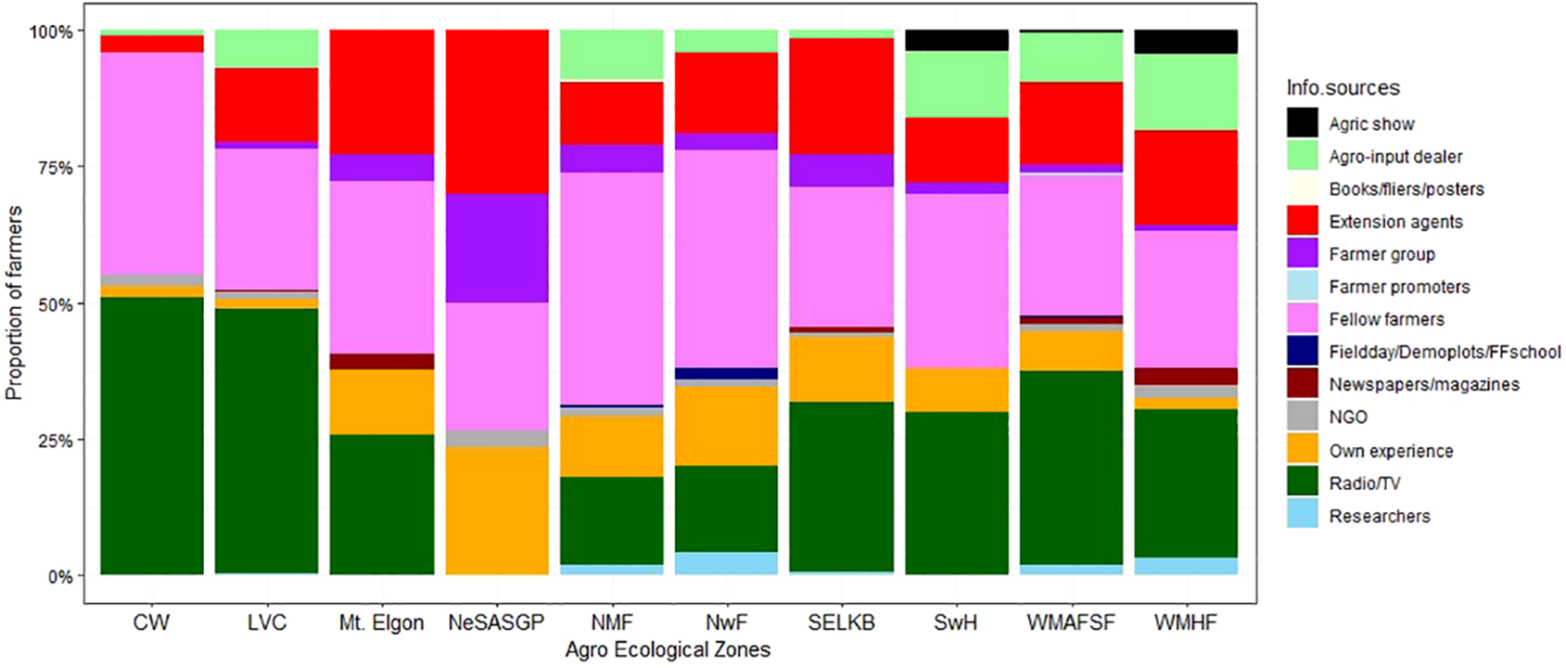
Proportion of farmers who received information on *Spodoptera frugiperda* from different sources in the different agro-ecological zones of Uganda, 2018.

During the *FAW* invasion, maize farmers in different AEZs received information from different sources (**Figure 9**). The most common source of *FAW* information was farmer-to-farmer exchange, followed by radio/TV, extension agents, and input dealers. In addition, the farmers’ exploration of their own experience was an important factor regarding *FAW*. Although to a very minimal extent, print media, research and NGO extension also contributed to providing information regarding *FAW*.

## 4 Discussions

*Spodoptera frugiperda* is a recent introduction to Africa and has caused panic among different maize stakeholders. Because of the differences in ecological and probable farmer interventions, we conducted a study in different agro-ecological areas to document i) major constraints faced by farmers, ii) farmers’ awareness and knowledge of *FAW*, iii) maize grain yield losses, iv) practices used by farmers to control *FAW* and their perceived effectiveness; v) information sources on *FAW.* Our results revealed that most of the farmers were men and largely youths. Farmers reported drought, insect pests and labour shortage as major constraints faced in the different AEZs. Among the pests, *FAW*, stem borers, termites, and monkeys were the key ones affecting maize production in Uganda. All the respondents were affected by *FAW*, starting at different times. The major measure deployed by farmers in *FAW* management was the use of synthetic insecticides. During the study period, farmers mainly received information on *FAW* from Radio/TV, fellow farmers, extension agents, and their own experience. The greatest proportion of respondents being men in this study is consistent with previous findings from several other African countries, including Burkina Faso, Benin, Gabon, Mozambique, Senegal, Zambia and Zimbabwe (22–25). The dominance of men in Agriculture is largely attributed to their roles as household heads, landowners and key decision-makers (26). This does not negate the fact that men and women may play different roles in crop cultivation activities, as reported in Uganda (27). Moreover, men are readily available whenever there are interviews or group meetings. The dominance of most farmers with primary and secondary education is also another factor that might affect their responses to *FAW*. In this study, no significant associated between key variables of interests were found.

The production constraints reported by the farmers in this study to be affecting maize production are facts that are consistent in many maize farming systems in Africa. Drought, insect pests, labor constraints, poor soils and unpredictable rainfall patterns were the key constraints among the maize farming communities in this study. Similar observations were made in Ghana (28) and Zambia (24). This clearly is an indication that smallholder farming systems are affected by various production constraints, categorized as abiotic, biotic and socio-economics. These constraints need to be addressed holistically in order to realize good yields and make maize farming more profitable. Among the pests, *FAW* was ranked as the most important pest of maize. This may be attributed to the rapid spread and extensive damage caused by the pest. The number of farmers who reported stemborers as a problem was lower than those for *FAW*. This may partly be because of the high reproductive rate and aggressive nature of *FAW*, leading to higher damage incidence and more severe symptoms than those of stemborers. However, a team from Cameroon concluded that there is spatial-temporal partitioning of the two pests following a few cases of co-occurrence of *FAW* and stemborers observed at the later maize growth stage in Cameroon (29). Indeed, the research team noted a low occurrence of stemborers and some form of co-occurrence in a lower number of plants and fields. The difficulty in differentiating early damage symptoms of stemborers from those of *FAW* and the subsequent entry of stemborer larvae into the maize stems could partly have resulted in a lower number of farmers reporting it as a pest during the study period. Termites were also listed as a problem in maize production. This is an emerging problem that may negate the gains from *FAW* control as they cut down mature plants. They ring bark young crops or cut the stems of maize plants, leading to death (30). Among the primates, monkeys were reported as pests of maize in some AEZs. Indeed, monkeys are primates responsible for the human-wildlife conflict, and affecting maize in Nepal and Ethiopia mainly near the wildlife and or forested areas (31–33). The range of pests, therefore, calls for a comprehensive maize pest management package that takes into account all economic pests that occur in an area.

Contrary to the reported first occurrence of *FAW* in Africa and in Uganda in 2016, results from this study indicate that some farmers first saw *FAW* in 2014 and 2015. Other studies that documented farmers’ observations of *FAW* also indicate that the pest was seen well before its first official report, with some farmers in Eastern Uganda reporting first notice in 2013 (18). On the other hand, farmers in Burkina Faso, DRC, Gabon and Senegal reported first noticing *FAW* in 2015 (25, 34). These observations point to the fact that *FAW* entered the countries earlier and had to first build up a sufficient population before causing substantial damage for it to be noticed. In fact, the period of notice in Uganda coincided with a period of drought that was associated with higher temperatures. High temperatures are known to favor rapid reproduction in *FAW* (35).

Studies conducted in Burkina Faso (34), Ethiopia and Kenya (36), Mozambique (23), and Zambia (24) indicated that most farmers were aware of the occurrence of *FAW* signs and symptoms in maize fields. In this study, most farmers could readily identify leaf damage symptoms and the presence of young and older larvae on maize plants. This may be because of the conspicuous nature of the two. The eggs and adults were rarely reported, perhaps due to limited knowledge of the biology and behavior of the pest; egg placement on the lower surface of the leaves, and adults hiding in the funnel of maize plants during the day. Another compounding factor is the occurrence of lower numbers of both egg and adult stages on maize plants as was observed by the team in the field and subsequent studies. Similarly, damage on the ears of maize plants was reported by a small fraction of farmers, and could relate to the low occurrence of ear damage in fields.

Maize yield loss due to *FAW* fell between 9% to 67% across the different seasons of Uganda. These losses fall within the ranges reported for *FAW* in several African countries. For instance, losses of 40% (range 25–50%) and 45% (range 22–67%) were reported for maize in Ghana and Zambia, respectively (16, 36). However, the study did not isolate the effect of drought and other key constraints reported by farmers as affecting maize production. Thus, the actual losses attributable to *FAW* may be lower than what is calculated in this study. Nevertheless, unpublished experimental studies within the country have demonstrated that yield losses attributable to *FAW* range from zero % to about 52% when the untreated control is compared with treated plots (Otim et al. unpublished). Variations in losses are associated with management practices and climatic factors such as variations in precipitation, temperature, and humidity which significantly impact *FAW* reproduction, spread and yield impact (37).

The application of synthetic pesticides was the most common method used by farmers to control *FAW* in Uganda. This is because the Government based on researchers’ recommendations supported the purchase and distribution and popularized the safe use of selected mainly synthetic pesticides for an emergency response to this problem (12), especially since it was a new pest. Furthermore, synthetic insecticides, especially organophosphates and pyrethroids, are readily available to farmers when needed. The predominance of insecticide application for *FAW* management was also reported in Benin, Botswana, Ethiopia, Ghana, Kenya, Tanzania and Zambia (34, 36, 38–43). Despite the Government effort to train farmers on safe use of pesticides, farmers did not necessarily consider efficacy, dosage and application timing (data not presented). This has serious implications for the health of users, consumers, the environment and the profitability of maize production. Consequently, the country has embarked on a number of interventions to develop a sustainable solution to the pest, including harnessing biological control into integrated pest management strategy (44) and training farmers and extension officers on the safe use of pesticides.

Although farmers use insecticides as the main management practice, studies from Ghana and Zambia (17, 28) demonstrated the complementary effect of insecticides and other control practices on maize grain yield. This highlights the need to develop an Integrated Pest Management strategy for *FAW*, which incorporates cultural practices, biological control and decision tools for pesticide use. The IPM decision tool depends on established Economic Thresholds that are determined for pests under different production scenarios. In the case of *FAW*, and consistent with our study, it is evident that losses increase with an increase in *FAW* damage severity. This emphasizes the need for a decision tool to initiate the use of pesticides. Unfortunately, economic injury levels and economic thresholds (ETs) to control *FAW* have not yet been determined in Uganda. Consequently, the country scientists recommend ETs, which are used in the Americas, or blanket recommendations.

Access to relevant information is crucial for managing pest problems in farming systems. Radio/Television, fellow farmers, extension officers and agro-input dealers were the main sources of information on *FAW*. This is consistent with the reports on information sources in Zambia (38). This is because the Government, research, extension and NGOs mainly used these different pathways to reach the farmers at the peak of the epidemic. These highlight the need to equip farmers, extension officers and agro-input dealers with the right information for better sensitization of farmers either directly or through media.

## 5 Conclusions

This study established that maize farmers face several challenges, including insect pest problems. Among the maize insect pests, the *FAW* has become the most damaging in recent years. All farmers were aware of and had experienced *FAW* damage in their fields by the time of the study. There were, however, cases of farmers reported not seeing the eggs and FAW moths. Estimated yield losses (not disaggregated by cause) varied between seasons and AEZs and ranged from 9.4% in WMAFSF in 2016B to 66.8% in NwF in 2017B. In response to the emergence of the pest, farmers adopted various methods of management. The application of synthetic pesticides was the most common method used by farmers to control *FAW* in Uganda. However, the use of insecticides in maize fields did not necessarily take into account efficacy, dosage and application timing (data not presented), which has serious implications for the health of users, consumers, the environment and the profitability of maize production. The farmers sourced information on *FAW* from various sources, including farmer exchange, radio/TV, extension agents, input dealers, print media, research and NGO extension.

Basing on the findings of this study and other related researches in Uganda, there is a need for more targeted sensitization to create awareness and knowledge on the biology and ecology of *FAW*, and the benefits and drawbacks associated with pesticide use. The promotion of control should be targeted to specific ago-ecologies and under given environmental conditions. The main sources of information for farmers (farmer to farmer exchange, radio talk shows and extension agents) should be used to channel production information timely and effectively. This should be preceded by packaging clear, concise and uniform information for the farmers and other stakeholders.

The current package recommended to control *FAW* in Uganda includes early planting, proper weeding, fertilizer application, intercropping with legumes, handpicking or ploughing to expose pupae, and judicious application of biopesticides or synthetic pesticides. While these are key tactics in managing *FAW*, there are information gaps that are being addressed to come up with a more well-researched and comprehensive *FAW* management package. In studies (data not presented), we found that although pesticide application reduces *FAW* infestation and damage, it does not always result in added yield advantage over the untreated control, especially when rainfall is high. We therefore recommend an integrated crop management strategy, which incorporates good agronomic practices such as early planting, fertilizer application, proper weeding, and scouting and applying pesticides based on infestation/damage thresholds.

On the research front, there is a need develop and promote a sustainable solution for *FAW* management, including harnessing biological control, appropriate agronomic practices and other relevant cultural methods instead of relying on synthetic pesticides. The ongoing research efforts are seeking to; 1) monitor the distribution and severity of *FAW* damage, 2) assess grain yield benefits associated with *FAW* damage, 3) characterize insecticide resistance profiles of different *FAW* populations, 4) harness the use of cultural and natural controls, 5) evaluate the cost–benefit of different insecticides and spray regimes, and 6) evaluate the impact of different classes of insecticides on *FAW* natural enemies, among others. These together with the information generated earlier will foster the development of more sustainable *FAW* management options.

## Data availability statement

The raw data supporting the conclusions of this article will be made available by the authors, without undue reservation.

## Author contributions

TO: Formal analysis, Visualization, Writing – original draft, Writing – review & editing. IO: Formal analysis, Investigation, Methodology, Writing – original draft, Writing – review & editing. RA: Investigation, Methodology, Writing – review & editing. SA: Conceptualization, Funding acquisition, Investigation, Methodology, Supervision, Writing – review & editing. SA: Investigation, Writing – review & editing. DO: Formal analysis, Visualization, Writing – original draft, Writing – review & editing. BR: Writing – original draft, Writing – review & editing. MO: Conceptualization, Formal analysis, Funding acquisition, Investigation, Methodology, Project administration, Resources, Supervision, Visualization, Writing – original draft, Writing – review & editing.
